# Are We Truly Addressing the Elective Surgery Backlog?

**DOI:** 10.7759/cureus.103521

**Published:** 2026-02-13

**Authors:** Daniel Jones, Maulik Gandhi

**Affiliations:** 1 Trauma and Orthopaedics, Bradford Royal Infirmary, Bradford, GBR; 2 Orthopaedics, Bradford Royal Infirmary, Bradford, GBR

**Keywords:** accurate data, backlog, coding, elective surgery, waiting time

## Abstract

Background

Orthopaedics currently has the largest waiting list of any surgical speciality in the UK, and the number of patients awaiting elective surgery is continuing to climb. This retrospective study aims to quantify the impact of coding errors on elective surgery waiting times at a large UK district general hospital.

Method

Data from 381 patients who underwent operations on elective orthopaedic operating lists between January 1, 2025, and March 31, 2025, were included in the study. Most orthopaedic patients with acute issues are operated on a dedicated acute list. However, surges in acute referrals may result in re-purposing of elective operating lists to treat acute patients. These cases are at risk of being incorrectly coded as elective operations. The electronic booking code of the operation was compared with the case notes of each patient to determine if the coding as elective or acute was accurate.

Results

Of the 381 patients coded as elective, 44 were acute cases falsely coded as elective. The mean waiting time for these false elective cases was 10.86 days, in comparison to 247.24 days for the true elective cases. Overall, incorrect coding of trauma cases as elective cases artificially reduced the mean waiting time by 27.14 days, or 11.01%. A Mann-Whitney U test showed this to be statistically significant (p = 0.012).

Conclusion

Inaccurate electronic coding substantially and artificially reduces orthopaedic surgery waiting time. This is likely driven by human error and software errors that can prevent correct coding. These issues might be addressed through a review of the approved Electronic Patient Record (EPR) software systems, staff training, and a further push towards cold-site operating to better separate acute and elective orthopaedic care. Finally, a multi-centre study would demonstrate how far-reaching these coding errors are.

## Introduction

Inaccurate electronic coding of surgical operations may be masking an even greater backlog than suggested by the current data. Following the COVID-19 pandemic, the number of patients in the UK waiting over one year for elective surgery hit an all-time high at 436,127, accompanied by a total waiting list of 4,950,297 [1]. Since then, the number of those waiting over a year has fallen to 191,493, though the total number on elective waiting lists has risen to 7,414,794 [2]. Orthopaedics currently has the largest waiting list of all surgical specialities, at over 800,000 patients, of which 27,000 have been waiting more than one year [2].

This study aims to quantify the impact of coding errors on elective waiting times at a large UK district general hospital, where elective and acute orthopaedic operations are carried out at the same site. In this hospital, there is a dedicated theatre list for acute orthopaedic operations. However, surges in trauma cases regularly result in utilisation of elective orthopaedic lists for treatment of acute orthopaedic patients. Once an acute patient is assigned a slot on an elective list, their operation may be incorrectly coded as an elective case; this results in an artificial reduction in elective surgery waiting times. 

## Materials and methods

Study design

This study is a retrospective analysis of patients who underwent surgery on elective orthopaedic operating lists between January 1, 2025, and March 31, 2025. This three-month period was selected to account for some of the month-by-month variation in both acute referrals and elective operating and covers both winter and early spring, when seasonal pressures might vary. A total of 381 patients were included in the final analysis; two patients were excluded due to incomplete waiting time data. At times, acute patients may be operated on elective lists; these cases are at risk of being incorrectly coded as elective. Acute cases were defined as those that were referred through acute pathways, discussed in an acute referrals multi-disciplinary meeting, and documented as appropriate for acute orthopaedic care. Conversely, elective cases were defined as those referred through elective pathways, reviewed in an elective clinic, and documented as appropriate for elective care. The electronic booking code of the operation was compared with the case notes of each patient to determine if the coding was accurate. There are likely two reasons for this incorrect coding. Firstly, if an acute patient is designated as ‘walking wounded’ and can safely await an operation date at home, a design flaw in the hospital system prevents those patients who are at home from being booked and coded as an acute case until they return to the hospital. Secondly, human error allows staff to incorrectly book trauma cases as elective.

Data collection

All patients referred for acute orthopaedic input are logged on an electronic referrals form. The data from this was cross-referenced with the Electronic Patient Record (EPR) to confirm the following data points: firstly, that the acute operation was carried out on an elective list and not an acute list; secondly, whether the operation was booked and coded as acute or elective. Finally, to calculate waiting time, the dates of the operation booking and the operation itself were logged. Over the same three-month period, the booking date and operation date for all elective cases were also recorded.

Data analysis

Waiting time data, expressed as calendar days, was subdivided into three groups. Firstly, a true elective group included those patients who underwent elective orthopaedic procedures on elective lists. A false elective group included patients who underwent acute operations on elective lists but were incorrectly coded as elective. Finally, an unfiltered elective group combined data from both the true and false elective groups. 

Microsoft Excel (Microsoft® Corp., Redmond, WA, USA) and StatsKingdom software were used for data analysis. Levene’s test was used to establish the equality of variance between the true elective and unfiltered elective groups. The D'Agostino-Pearson test established if the data sets followed a Gaussian distribution. Finally, the significance of variation between the true elective and unfiltered groups was assessed with the Mann-Whitney U test.

## Results

Of the 381 patients coded as elective, 44 were acute cases that were falsely coded as elective, leaving 337 true elective cases. The mean waiting time for false elective cases was 10.86 days, and 247.24 days for true elective cases. When combined as the unfiltered elective group, the mean waiting time fell to 219.94 days. Overall, incorrectly coding trauma cases as elective cases artificially reduced mean waiting time by 27.14 days, or 11.01% (Table 1). 

**Table 1:** Descriptive statistics for the data set

Measure	Value
Total false elective cases	44
Total true elective cases	337
Total unfiltered elective cases	381
Percentage of unfiltered cases that were false elective cases	11.54% (n = 44)
Mean waiting time for false elective cases (days)	10.86
Mean waiting time for true elective cases (days)	247.24
Mean waiting time for unfiltered cases (days)	219.94
Percentage reduction in elective waiting time due to incorrect coding	11.01% (n = 27.14 days)

The statistical significance of this fall in waiting time was determined as follows. Levene's test demonstrated no significant difference in variance (p = 0.364). The distribution of the unfiltered elective and true elective groups was both substantially and significantly non-Gaussian. Therefore, non-parametric testing was appropriate. A Mann-Whitney U test found the difference in waiting times to be significant (p = 0.012, Table 2).

**Table 2:** Statistical analysis of the data set

Statistical test	Value
True elective: D'Agostino-Pearson test of skewness and kurtosis	p = 0.00004863
Unfiltered elective: D'Agostino-Pearson test of skewness and kurtosis	p = 0.000002415
Levene's test of equality of variance	p = 0.364
Mann-Whitney U test	p = 0.012

## Discussion

The key finding from these data is that, in this hospital, poor coding practices artificially reduce mean elective orthopaedic waiting times by almost one month (27.29 days) or 11.04%. This reduction in waiting time was statistically significant. The wider implication of this may be a substantial underestimation of the orthopaedic surgery backlog. This, in turn, may lead to underestimation of key resources, such as theatre staff, clinic appointments, and operating theatres, that are required to tackle these long waiting lists.

Considering the impact that falsely low waiting times may have on patients, the NHS Choose and Book service allows patients to select a hospital of their choice for many elective procedures. Artificially shorter waiting times might unfairly draw patients to a particular hospital. However, the degree to which patients consider performance metrics is perhaps not so substantial. The King’s Fund Patient Choice report found that leaflets on hospital performance and the NHS Choices website were used by 6% and 4% of patients, respectively. Instead, 41% relied on prior experience, 36% used advice from their general practitioner (GP), and 10% looked to friends and family [3].

There are likely two main driving factors for this coding inaccuracy. Firstly, a software error can prevent patients from being coded as having an acute operation unless they are formally admitted to the hospital system. The second is human error - not recognising acute and elective orthopaedic operations and coding inappropriately as a result. Determining the proportional role of each might be achieved through a root-cause analysis.

Though this is a single-centre study, the issue may be widespread. NHS England reported that, as of December 2023, 90% of NHS trusts used an EPR [4]. Currently, NHS England has approved eight different suppliers of EPR software systems, though a breakdown of the utilisation of each company’s product is not publicly available [5]. Consequently, how far-reaching this software issue is remains unclear.

One might argue that coding errors could be reduced with a single bespoke system that allows system flaws to be quickly addressed and fail-safe mechanisms to be built in. While some trusts do have bespoke in-house software, perhaps with greater control of functionality [6], this idea is unlikely to gain much traction as it is at odds with current NHS policy. The present drive from NHS England is to better integrate these different software systems through the NHS Federated Data Platform project, rather than implementing a single nationalised EPR [7]. Perhaps what is needed is a review of the fitness-for-purpose of the eight EPR software suppliers.

Considering the human error element, early research into improving the accuracy of hospital coding showed that greater clinician involvement achieved greater accuracy. Yeoh and Davies noted an increase in the coding accuracy of diagnoses and procedures from 54% to 85% following transfer of coding responsibility to clinical staff [8]. Similarly, Nouraei et al. found 24.1% of otolaryngology diagnoses to be incorrect, and implementation of a clinician-coder multidisciplinary team (MDT) was both cost-effective and resolved these inaccuracies [9]. In this unit, the theatre bookings are made by clinicians or theatre coordinators familiar with acute and elective orthopaedic procedures. Therefore, clinician involvement as a method of accuracy improvement is already addressed to some extent. Staff interviews to identify gaps in knowledge and additional training might enhance this further.

Another substantial problem potentially driving this issue is the lack of cold-site operating in this NHS trust. Cold-site operating is the practice of carrying out elective orthopaedic work in a dedicated building, separate from acute trauma. It follows logically that, if acute and elective operations are kept separate, the risk of acute orthopaedic surgery being coded as elective through human error should be reduced. The Getting It Right First Time (GIRFT) report showed that cold sites reduce elective orthopaedic length of stay, reduce day-of-surgery cancellations, and increase the number of operations performed [10]. The findings in this project suggest that an improvement in the accuracy of waiting time data might also be an additional benefit of cold-site operating.

Inaccurate data from poor coding is not exclusive to orthopaedic surgery waiting times. A review of 712 patients undergoing cholecystectomy found that 172 operations were coded as open when only 58 operations truly were. This data resulted in the trust being identified as having a comparatively high rate of open cholecystectomy and as performing poorly. Following case note review and generation of accurate data, the trust was shown to be performing well, with a statistically low open cholecystectomy rate [11]. Similarly, a meta-analysis examining the correct coding of diagnoses in NHS discharge letters found that there was a substantial difference between the coded diagnosis and the diagnosis referenced in case notes. Median accuracy was 83.2%, with a wide inter-quartile range of 67.3%-92.1% [12]. Steps have been taken nationally to try to improve the accuracy of coding. NHS England has established the Data Quality Assurance Framework, which aims to push excellence in data collection accuracy and looks to resolve data quality issues [13]. It is possible that other national frameworks might disincentivise more accurate data. For example, the shorter waiting times of incorrectly coded trauma cases will artificially improve waiting times and might boost a hospital’s standing in the new NHS league table [14].

Limitations

As touched on earlier, there is no publicly available breakdown of the eight different private EPR software companies. So the external validity of this data is not certain, though this could be addressed through a multi-centre study. Similarly, human error is likely a large component of incorrect coding; the proportion that this contributes is unclear in the absence of a formal root-cause analysis. Finally, this data was collected during a UK winter and early spring. One might postulate that, with icy and wet conditions, surges in trauma and subsequent re-purposing of elective operating lists may be more likely. Twelve months of data, to account for all seasonal fluctuations in trauma, would help to address this issue. 

## Conclusions

Despite the GIRFT report recommendations, balancing elective orthopaedic and trauma operating remains a significant challenge. The coding practices in this centre artificially and significantly reduce elective waiting times by 27.14 days, or 11.01%. This masks the true scale of the elective backlog, the volume of trauma operating, and may prevent a proportional response to the issue. Review of the approved EPR software providers might help improve software issues, and cold-site operating would also likely improve the accuracy of coding. A detailed root-cause analysis may help tease out how software and human error each contribute, and staff interviews regarding accurate coding could form a basis for training content. Finally, a regular audit of coding accuracy should be considered to promote sustainable change. Fundamentally, the current orthopaedic backlog reflects a system that has too few resources for the demands placed upon it, and inaccurate data is likely hindering the ability to tackle it.
